# Clinico-Demographic Profile of Children and Adolescents with Attention Deficit Hyperactivity Disorder Presenting to a Tertiary Care Centre: A Descriptive Cross-sectional Study

**DOI:** 10.31729/jnma.8539

**Published:** 2024-04-30

**Authors:** Utkarsh Karki, Supriya Sherchan, Anil Sharma, Amit Jha

**Affiliations:** 1 Child and Adolescent Psychiatrist, Child and Adolescent Psychiatry Unit, Kanti Children's Hospital, Kathmandu, Nepal; 2 Psychiatrist, Child and Adolescent Psychiatry Unit, Kanti Children's Hospital, Kathmandu, Nepal; 3 Clinical Psychologist, Child and Adolescent Psychiatry Unit, Kanti Children's Hospital, Kathmandu, Nepal

**Keywords:** *ADHD*, *adolescents*, *children*, *comorbidity*, *medication*

## Abstract

**Introduction:**

Attention deficit hyperactivity disorder (ADHD) is a common neurodevelopmental disorder in children. ADHD leads to significant impairment in overall functioning of the child. There is limited information concerning the clinical scenario of ADHD within Nepal. The study aims to determine the clinico-demographic profile and pattern of medication use in the treatment of ADHD.

**Methods:**

This study retrospectively examines the records of children diagnosed with ADHD at the Child and Adolescent Psychiatry (CAP) Unit, Kanti Children's Hospital (KCH), Nepal. Approval for the study was granted by KCH's Institutional Review Board. The analysis focused on data extracted from hospital records of ADHD patients spanning from 1 January 2021 to 30 June 2023 encompassing two and a half years.

**Results:**

A total of 585 children were diagnosed with ADHD, with a mean age 7±3.04 years. The majority 501 (85.64%) were male, and 377 (64.44%) were from the school going age group (6 to 11 years). The prevalent psychiatric comorbidities included Autism Spectrum Disorder (ASD) at 102 (17.43%), Intellectual Disability (ID) at 93(15.89%), and Oppositional Defiant Disorder (ODD) at 36 (6.15%). The commonly used medication was Clonidine 165 (28.20%) followed by Atomoxetine 154 (26.32%) and Risperidone 65 (11.11%).

**Conclusions:**

The study indicates that ADHD is highly prevalent in Nepal. Comorbidities like ASD and ID are frequently seen which further necessitates the need for structured assessments and multidisciplinary approaches to address ADHD. In our context with limited treatment options, the management of ADHD is extremely challenging.

## INTRODUCTION

Attention Deficit Hyperactivity Disorder (ADHD) is a common childhood neurodevelopmental disorder characterized by age-inappropriate levels of inattention, and or impulsivity and hyperactivity. Meta-analyses have estimated the prevalence of ADHD in children and adolescents (CAA) as 5.29%.^1^ Boys are diagnosed more with ADHD than girls with ratio of 3:1.2 ADHD is associated with various comorbidities in the range from 67-87% in clinically referred samples.^3^

Children with ADHD have detrimental impact on their social, academic, psychological functioning. Despite being very common, limited data are available regarding ADHD in Nepal. ADHD is a treatable condition, and despite robust evidence based treatments available, we do not have studies showing what treatment approaches are carried out in our country.

The study aimed to explore the clinico-demographic profile and pattern of medication use in CAA with ADHD presenting to the Child and Adolescent Psychiatry (CAP) Unit of a tertiary care centre.

## METHODS

This is a retrospective chart review of ADHD patients registered between 1st January 2021 to 30th June 2023. The study was conducted in the CAP unit of Kanti Children's Hospital Kathmandu, Nepal which is a tertiary care centre in Bagmati province (i.e. province 3). Ethical approval was obtained from the Institutional Review Board (IRB) (Reference No: 15). All recorded cases were included in the study to analyze the clinical profile. Data was collected from the register which was maintained in the CAP unit of Kanti Children's Hospital The register book had the following information recorded:name, age, gender, address, diagnosis, treatment. All cases had undergone a detailed clinical evaluation based on unstructured psychiatric interviews with both the child and parent/s by a child and adolescent psychiatrist or psychiatrist followed by a clinical psychologist. Consent was not applicable as this was retrospective study; however, confidentiality of data was maintained. For analysis of data, the age group was categorized as pre-school (less than 6 years), school going (6-11 years) and adolescent (12-18 years). As the hospital tertiary care centre, patients from all seven provinces are referred or come for treatment to this hospital, therefore patient populations were also categorized according to provinces. Data on comorbidity and patterns of medication use was also recorded. Data was entered and analyzed in Microsoft Excel. Descriptive statistics was used to calculate frequency and proportion.

## RESULTS

A total of 14,443 patients attended the out-patient services of CAP unit during this two and a half years period. We identified 585 (4.05%) patients with a diagnosis of ADHD in the records. The data was not skewed. The mean age of children with ADHD was 7±3.04 years and among them 501 (85.64%) were boys. The children in the school going age group were 377 (64.44%) followed by preschool age group 133 (22.73%) and adolescent group 75 (12.82%). There were 425 (72.64%) patients from Bagmati province (Table 1).

**Table 1:** Socio-demographic profile of patients with ADHD (n= 585).

Characteristics		n (%)
Gender	Male	501 (85.64)
	Female	84 (14.35)
Age group	Preschool (<6 years)	133 (22.73)
	School going (6-11 years)	377 (64.44)
	Adolescent (12-18 years)	75 (12.82)
	1	41 (7)
	2	20 (3.41)
	3	425 (72.64)
	|4	45 (7.69)
	5	35 (5.98)
Provinces	6	5 (0.85)
	7	14 (2.39)

There were 361 (61.70%) patients with comorbid condition. Out of the total cases, 269 (45.98%) were found to have one comorbidity, 82 (14.01%) had two comorbidities and 10 (1.70%) had three comorbidities (Table 2).

**Table 2:** Pattern of comorbidities ADHD (n = 585).

Comorbidity	n (%)
Absent	224 (38.29)
Present	361 (61.70)
With one comorbidity	269 (45.98)
With two comorbidities	82 (14.01)
With three comorbidities	10 (1.70)

The most common psychiatric co morbidities were Autism Spectrum Disorder (ASD) 102 (17.43%) followed by Intellectual Disability (ID) 93 (15.89%) and Oppositional Defiant Disorder (ODD) 36 (6.15%) (Table 3).

**Table 3:** Types of comorbidities in patients with ADHD (n= 585).

Comorbidities	n (%)
Autism spectrum disorder	102 (17.43)
Intellectual disability	93 (15.89)
Epilepsy	39 (6.66)
Oppositional defiant disorder	36 (6.15)
Anxiety disorder	28 (4.78)
Language disorders	22 (3.76)
Conduct disorder	18 (3.07)
Major depressive disorder	12 (2.05)
Borderline intellectual functioning	10 (1.70)
Specific learning disorder	10 (1.70)
Word sound disorder	6 (1.02)
Tic disorder	6 (1.02)
Downs syndrome	5 (0.85)
Obsessive compulsive disorder	5 (0.85)
Global developmental delay	3 (0.51)
Hearing impairment	3 (0.51)
Tuberous sclerosis	2 (0.51)
Gratification disorder	2 (0.51)
Childhood onset fluency disorder	1 (0.17)
Xeroderma pigmentosa	1 (0.17)

There were 388 (66.32%) patients on medications, the remaining 197 (33.67%) exclusively received non-pharmacological intervention in the form of psychoeducation, environmental modifications, behavior therapy and parent management training. Out of the total sample, 388 children with ADHD were on medications. The commonly used medication was Clonidine 165 (28.20%) followed by Atomoxetine 154 (26.32%) and Risperidone 65 (11.11%). There were 11 (1.88%) children on a stimulant i.e. Methylphenidate. Bupropion, Modafinil and Imipramine were the offlabel ADHD medications used. (Table 4).

**Table 4:** Pattern of medication use in patient with ADHD (n = 585).^*^

Medications	n (%)
Without medication With medication Clonidine Atomoxetine Risperidone Modafinil Methylphenidate Imipramine Bupropion	197 (33.67) 388 (66.32) 165 (28.20) 154 (26.32) 65 (11.11) 15 (2.56) 11 (1.88) 4 (0.68) 3 (0.51)

* Multiple Response Question

## DISCUSSION

The differences could be due to differences in the number of sample size, hospital versus community study design done in the primary school children. The study done by Rimal and Pokharel had 41 children with ADHD out of 350 children in a 1 year period.^4^ However, our study had a total of 14,443 patients visiting to the only tertiary children's hospital of Nepal in the two and a half year period. This large number of total patients presenting to our centre could have resulted in lower prevalence of ADHD compared to other study done in Nepal.^4^ The other reason for higher prevalence of ADHD in the study done by Rimal and Pokharel could be due to the use of structured rating scale that was not done in all of the patients presenting to us.^4^ The diagnosis of ADHD in our study sample was primarily based on detailedpsychiatric interview and clinical assessment. This may have resulted in under diagnosis and missed diagnosis of ADHD in our study. The sample was over represented by boys 501 (85.64%). In a similar Italian register based study,85% of the ADHD cases were found to be of male gender.^6^ From our study, male to female ratio was found to be 5.9:1. Reported male to female ratio varies considerably from 2:1-10:1.7 Higher ratio of male to female was seen in clinic versus population based samples. ADHD is more common in boys as compared to girls due to biological differences, diagnostic biases, social and environmental factors and under diagnosis in females.^8^ Boys with ADHD present more with externalizing behavioral problems which is very obvious and troubling to their surroundings whereas girls with ADHD may attract much attention due toinattention presentation and internalizing problems. Hence, parents and teachers would not have sought consultation for girls. The mean age of children was 7 years which is similar to the findings of the study donein Nepal.^4^ More than half of the children 377 (64.44%) were of school going age group (6 to 11 years). Similar to our findings, a recent systematic review and meta analyses showed that children aged 3 to 12 years had higher prevalence of ADHD than adolescents.^9^ Another reason,why this age group presented more could be due to increasing school demands that ADHD got more attention.^10^ Comorbidity was found in 361 (61.70%) of our sample which is quite high and similar to international studies that have reported psychiatric comorbidities to range between 56 to 88% amongst the patient with ADHD. Studies from India have reported psychiatric comorbidity to range from 34.8% to 53%.^11,12^ The reasons for differences in rate of comorbidity include sample size, sample characteristics such as age, gender, study settings, screening instruments and socio-cultural background. The reason for differences could be due to more male gender presenting with ADHD, inclusion of all age group of below 15 yrs, the unstructured interview technique rather than the use of structured assessment tools. Studies have shown that ADHD comorbidity was present in 40-80% depending upon the sample. In clinically referred samples comorbidity can be high as 67-87% which is similar to our finding.^3^ The presence of co morbidities adds to the burden of illness and has an impact on the severity of ADHD. Hence, comorbidity needs to be explicitly looked for during evaluation and managed appropriately. The common comorbidity in our sample was ASD in 102 (17.43%), ID in 93 (15.89%) and ODD in 36 (6.15%) childrens. Neurodevelopmental disorders were found to be the commonest comorbidity in our sample which is in contrast findings from other studies.^4,12^ In line with our study, Jogia et al found that among 428 samples of ADHD, ASD was found to be amongst the top 5 comorbid disorders and in another study done in Nepal 12% of the sample had ASD as a comorbidity with ADHD.^13^ One of the commoncomorbidities of ID in our study was also reported in a retrospective chart review done in India.^10^ One of the reasons could be that most studies have excluded ID and ASD whileassessing psychiatric comorbidities in ADHD. It was surprising to see low rates of ODD, SLD, mood and other anxiety disorders and tic disorders in our study which is in contrast to previous studies.^4, 12, 14,15^ Treatment for ADHD in our study showed that most common medication used was Clonidine 165 (28.2%) followed by Atomoxetine 154 (26.3%) and Risperidone 65 (11.1%). A study done in India on preschool children with ADHD, the most commonly prescribed drug was Clonidine followed by Risperidone that is similar to our study.^16^ Recently, Atomoxetine was introduced in Nepal, following which there has been an ease and rise in the use of Atomoxetine. Guidelines and evidence recommend stimulants as the first line of medication for ADHD.^17^ However, Methylphenidate a stimulant was only prescribed for 1.8% (n=11) of the sample. The main factor limiting the use of stimulant is lack of availability in our country and presence of comorbid seizure disorder. The possible reasons for use of Risperidone in 11.1% of the sample could be impulsivity and aggression posing injury to self and others and easy availability. More prescription of Risperidone due to lack of availability of stimulants is a growing concern that needs to be addressed. Apart from pharamacotherapy, in our sample psychoeducation, behavior interventions, parent training and liaison with school was provided to all children and adolescents as a part of treatment of ADHD. The possible reasons for Clonidine as the commonly prescribed medications could be cost effectiveness, followed by the presence of seizure disorder which is a relative contraindication for Methylphenidate and need for sedative property. These insights into medication patterns emphasize the limitation of treatment options, with unavailability of stimulants and underline the pressing need to apprise concerned stakeholders and policy makers. The major limitation of the study is the retrospective design. Data regarding the type of presentation of ADHD were not mentioned in the register, hence type of ADHD presentation could not be analysed. Second,subjective errors during the register recordings may have occurred where comorbidities may have been under reported or over reported. Third, the presence of comorbidities was based upon clinical evaluation only with no support of standardized and structured instruments. Despite all the drawbacks, it is a comprehensive review of available clinical data on ADHD in children and adolescents.

## CONCLUSIONS

Our study shows ADHD is highly prevalent in Nepal. Comorbidities like ASD and ID are frequently seen with this condition which further necessitates the need for structured assessments and multidisciplinary approaches to address ADHD. In our context with limited treatment options, the management of ADHD is extremely challenging.
